# Role of metabolites in mediating the effect of gut microbiota on Crohn disease: A two-step Mendelian randomization (MR) study

**DOI:** 10.1097/MD.0000000000046253

**Published:** 2025-11-28

**Authors:** Heng Shi, Qin Peng

**Affiliations:** a Department of Gastroenterology, The Central Hospital of Shaoyang, Shaoyang, Hunan Province, China.

**Keywords:** Crohn disease, genus Ruminococcaceae UCG013, gut microbiota, Mendelian randomization, metabolites

## Abstract

This study aims to investigate the causal relationship between gut microbiota and Crohn disease (CD) and identify and quantify the role of metabolites as potential mediators. Using summary-level data from a genome-wide association study (GWAS), a two-sample Mendelian randomization (MR) analysis of genetically predicted gut microbiota and CD was performed. Furthermore, we used two-step MR to quantify the proportion of the effect of metabolites-mediated gut microbiota on CD. We identified 15 metabolites (carnitine levels, isovalerate (i5:0) levels, hydroxyoctanoate levels, 6-hydroxyindole sulfate levels, heptenedioate, 4-methylhexanoylglutamine levels, uridine levels, X-13866 levels, X-17354 levels, X-18345 levels, X-22834 levels, X-25519 levels, serine to pyruvate ratio, URIDINE to 2’-deoxyuridine ratio, and histidine to glutamine ratio) with mediating effects in the impact of genus Ruminococcaceae UCG013 on CD. Our study identified a causal relationship between gut microbiota and CD, with a small proportion of the effect mediated by fifteen metabolites.

## 1. Introduction

Crohn disease (CD) is characterized by chronic and recurrent inflammation of the intestinal mucosa. Its incidence and prevalence rates are increasing worldwide, and no cure exists. Although the exact cause is not yet clear, studies have shown that complex interactions between genetic susceptibility, environmental factors, microorganisms, and immune responses play a role in the occurrence and development of CD.^[1]^ Therefore, CD may result from the interaction of multiple factors. In recent years, gut microbiota has become a research hotspot and is considered to play an essential role in the progression of CD. The advancement of emerging science and technology has made metagenomics and metabolomics potential clinical research tools for exploring the correlation between gut microbiota metabolites and host mechanisms of action. Research has found that the metabolic profile of CD patients undergoes extensive changes, including alterations in short-chain fatty acids (SCFA) and bile.^[2,3]^ Metabolites of gut microbiota, such as acids, tryptophan, and succinic acid, can impact the body through various mechanisms.^[4]^

As researchers delve into the study of gut microbiota, they increasingly recognize the significant role of its metabolites as mediators in the interaction between gut microbiota and the host during the development of CD. These metabolites can be detected in various tissues, including feces, serum, urine, liver, cerebrospinal fluid, and intestinal tissue.^[5]^ A study^[6]^ conducted in the United States analyzed fecal non-targeted metabolomics and metagenomic sequencing data from 121 inflammatory bowel disease (IBD) patients and 34 healthy controls. The results revealed significant changes in the metabolic profile of IBD patients, with over 2700 metabolites identified, including various fatty acids, bile acids, amino acids (AAs), and sheath lipids. These metabolite changes were associated with IBD-related inflammation, suggesting that gut microbiota metabolites play a role in maintaining intestinal homeostasis. Current research indicates that the gut microbiota contains a wide variety of metabolites. These metabolites may affect the host’s complex physiological and pathological processes. It is essential to understand the metabolic signals between the gut microbiota and the host to uncover the development of Crohn disease.

Mendelian randomization (MR) is a powerful tool in epidemiological research that uses genetic variation to assess causal relationships between risk factors and specific diseases.^[7]^ Mediation MR evaluates the mediating role of a variable between exposure and outcome. One advantage is that MR is not affected by confounding factors, resulting in pure effects. Therefore, we aimed to assess metabolites’ role in mediating gut microbiota’s effect on CD.

## 2. Material and methods

### 2.1. Genome-wide association study (GWAS) data sources

The participants in the GWAS were of European ancestry, and all data used in our study were publicly available. We extracted GWAS data of CD from data freeze 10 of the FinnGen study. The dataset contained approximately 19 million single nucleotide polymorphisms (SNPs) associated with CD, comprising 2033 patients and 409,940 healthy controls (https://www.finngen.fi/en/access_results).

A GWAS data^[8]^ analysis of metabolites was performed on the Canadian Longitudinal Study on Aging (CLSA) cohort, which consisted of 8299 individuals of European descent. The study involved several large-scale GWASs that investigated 1091 metabolites and 309 metabolite ratios, publicly available in the GWAS Catalog (accession numbers from GCST90199621 to GCST90201020). Of the 1091 plasma metabolites tested, 850 belonged to 8 super pathways: lipid, AA, xenobiotics, nucleotide, cofactor and vitamins, carbohydrate, peptide, and energy. The remaining 241 metabolites were either unknown or only partially characterized.

The gut microbiota data^[9]^ used in this study was obtained from various groups of people with different racial backgrounds, ages, gender ratios, and microbiome analysis methods. The data included people from Europe, the Middle East, East Asia, Hispanic/Latin America, and Africa. The study focused on the European data (N = 14306) and found 226 different types of human gut microbiota, out of which 15 were unidentified. After removing the unidentified types, the analysis included 211 microbiotas.

All GWAS datasets used in this study were obtained from publicly available sources with preexisting ethical approvals. FinnGen study (CD data): Approved by the Coordinating Ethics Committee of the Hospital District of Helsinki and Uusimaa (Approval #HUS/1330/2020), with informed consent obtained from all participants. CLSA cohort (metabolites data): Received ethical approval from research ethics boards at all participating institutions across Canada, with documented written consent from participants. Gut microbiota GWAS: Approved by the Institutional Review Board of the University Medical Center Groningen (approval METc 2016/077), with informed consent obtained. As this study utilized summary-level GWAS data without individual identifiers, no additional ethical approval was required per institutional policies.

### 2.2. Selection of instrumental variables (IVs) and data harmonization

We selected SNPs that were genome-wide significant with a *P*-value less than 1 × 10^−5^. These SNPs were clustered based on linkage disequilibrium using a window size of 10,000 kb and *r*^2^ threshold of 0.001.^[10–12]^ To investigate the hypothesis of MR, we utilized the Phenoscanner database (https://maayanlab.cloud/datasets2tools/landing/tool/PhenoScanner) to ensure no associations between these SNPs and any known confounding factors.

To determine the strength of each IV, we used the *F*-statistic formula. The formula follows: *F* = *R*^2^ (N−2)/ (1−*R*^2^). Here, *R*^2^ represents the proportion of the variability of physical activity explained by each IV, and N is the sample size of the GWAS for the SNP-physical activity association. To calculate *R*^2^, we used the formula 2 × EAF × (1−EAF) × β^2^. Here, EAF represents the effect of allele frequency, and beta is the standard error of the genetic effect (Supplementary File S1, Supplemental Digital Content, https://links.lww.com/MD/Q797).

### 2.3. Primary analysis

**Figure 1.**
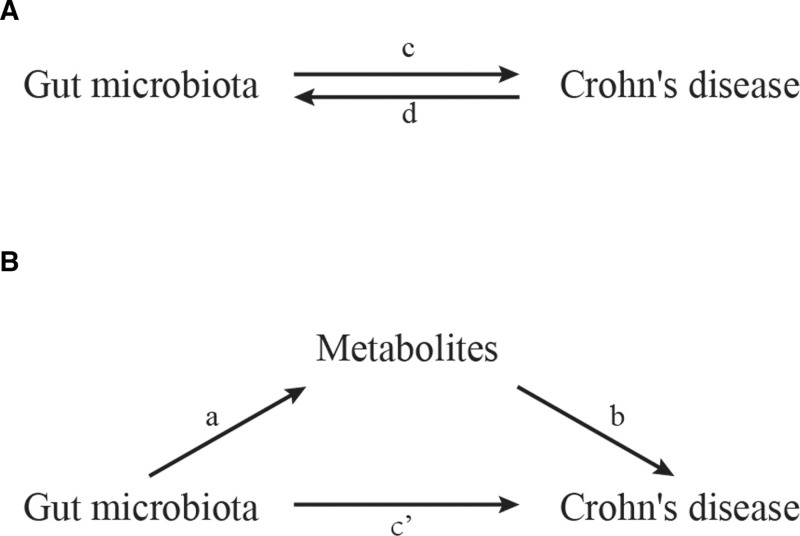
The diagrams illustrate the associations examined in this study. (A) “c” represents the total effect using genetically predicted gut microbiota as exposure and Crohn disease (CD) as an outcome. In contrast, “d” represents the total effect using genetically predicted CD as exposure and gut microbiota as an outcome. (B) The total effect is decomposed into (i) the indirect effect using a two-step approach, where “a” is the total effect of gut microbiota on metabolites, and “b” is the effect of metabolites on CD, and the product method (a × b), and (ii) the direct effect (c′ = c − a × b).

The analysis is summarized in Figure 1. We conducted a two-sample MR to evaluate the causality between gut microbiota and CD (Fig. 1A), designated as the total effect.

The inverse variance weighting (IVW) method uses Wald ratios to estimate the causal effects of each SNP, with MR-Egger and weighted-median methods as complements for robustness. IVW assumes all SNPs are valid instrumental variables (IVs). MR-Egger addresses directional pleiotropy, and the weighted median offers precision and consistency, even when up to 50% of the genetic variants are invalid IVs.^[13]^ Different methods cater to different validity assumptions for accurate MR estimates.

### 2.4. Mediation analysis

We conducted a two-step MR analysis to investigate whether metabolites mediate the causal pathway between gut microbiota and CD (Fig. 1). Gut microbiota affects CD through direct and indirect effects. The total effect includes direct effects (c’ in Fig. 1) and indirect effects mediated by gut microbiota through a mediator (a × b in Fig. 1). Mediated proportions were suppressed for metabolites where: The 95% confidence interval (CI) for β contained zero (no significant mediation), or The proportion calculation yielded negative values (indicating inconsistent mediation directions).

### 2.5. Sensitivity analysis

We conducted a sensitivity analysis to evaluate the reliability and robustness of the IVW method, which may be influenced by ineffective instrument bias or pleiotropy. We used Cochran *Q* statistic and its corresponding *P*-values to assess the heterogeneity of the selected IVs. To reduce the effects of horizontal pleiotropy, we employed the MR Egger intercept test and leave-one-out analyses. The significance of the intercept term indicated horizontal pleiotropy.^[14]^ In addition, scatter and funnel plots indicated the non-significant effect of outliers on the results and the strength of the correlation without heterogeneity, respectively.

### 2.6. Statistical analysis

All analyses were performed using R 4.3.0 software (http://www.rstudio.com). To conduct the MR analyses, we used the TwoSampleMR R package (version 0.5.7). The global-level testing was carried out at a significance level of a two-sided *P*-value of .05, and a *P*-value of less than .05 was considered statistically significant.

## 3. Results

### 3.1. The relationship between gut microbiota and CD

**Figure 2.**
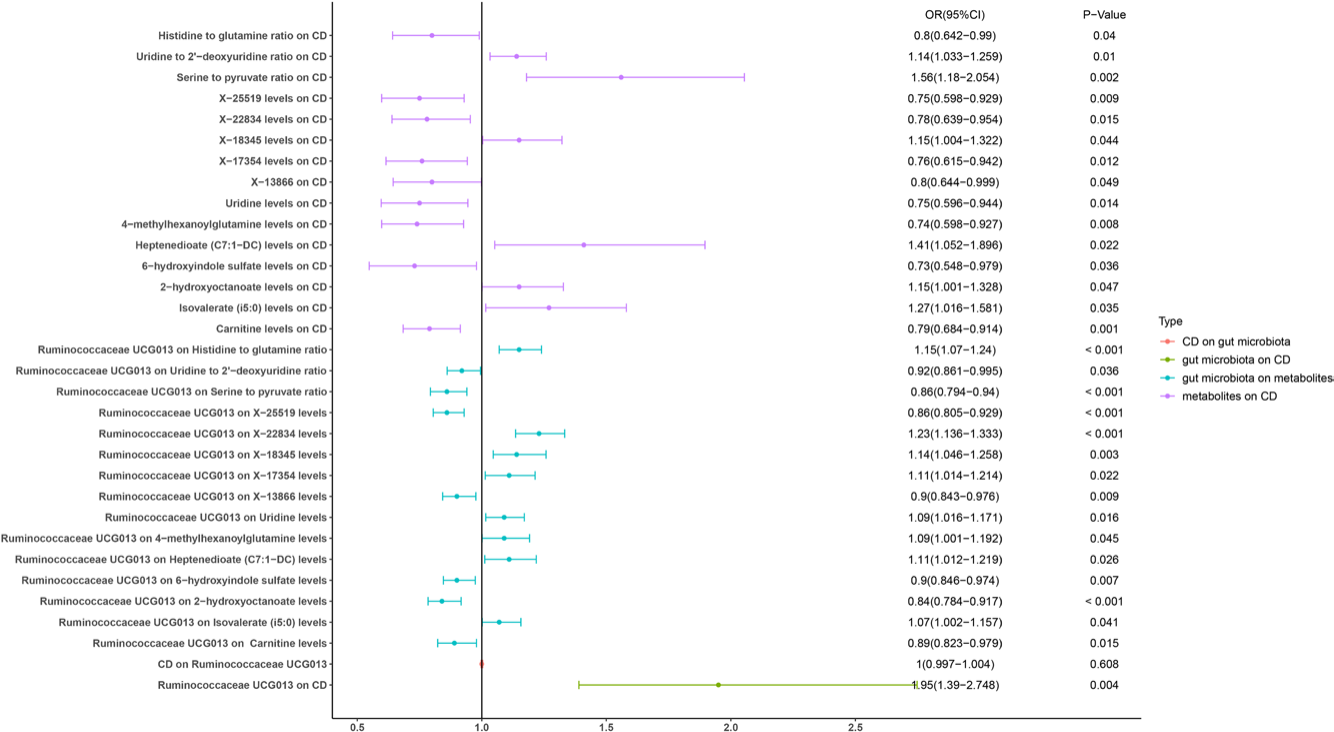
Forest plot to visualize the causal effects of metabolites with gut microbiota and Crohn disease.

Through our research, we identified a causal link between twelve specific types of gut microbiota and CD (Fig. 2): *Subdoligranulum* (odds ratio (OR): 0.65, 95% CI: 0.441-0.959, *P* = .029), *Adlercreutzia* (OR: 1.43, 95% CI: 1.047–1.948, *P* = .024), *Ruminiclostridium* UCG013 (OR: 0.60, 95% CI: 0.413–0.883, *P* = .009), *Ruminococcus* torques group (OR: 0.45, 95% CI: 0.280–0.731, *P* = .001), *Eisenbergiella* (OR: 0.77, 95% CI: 0.623–0.951, *P* = .015), *Bacteroides* (OR: 0.64, 95% CI: 0.429–0.945, *P* = .025), *Phascolarctobacterium* (OR: 0.67, 95% CI: 0.456–0.974, *P* = .036), phylum *Euryarchaeota* (OR: 1.18, 95% CI: 1.000–1.394, *P* = .047), order Bacillales (OR: 0.82, 95% CI: 0.682–0.989, *P* = .039), families Lachnospiraceae (OR: 1.39, 95% CI: 1.010–1.933, *P* = .044), and Bacteroidaceae (OR: 0.64, 95% CI: 0.429–0.945, *P* = .025) (Supplementary File S2, Supplemental Digital Content, https://links.lww.com/MD/Q797). Following these findings, we conducted a reverse MR analysis, which revealed a reciprocal causal relationship between CD and an expanded group of fourteen gut microbiota, encompassing the previously mentioned taxa along with the addition of Dorea and taxa from the orders NB1n and Verrucomicrobia, but not genus Ruminococcaceae UCG013 (Supplementary File S2, Supplemental Digital Content, https://links.lww.com/MD/Q797). To refine our understanding further, it is crucial to eliminate any potential confounding factors arising from mutual reverse causality between these microbiota and CD. In our subsequent analyses, we will include the genus Ruminococcaceae UCG013 to examine these complex interactions comprehensively.

### 3.2. The relationship between gut microbiota and metabolites

We have found significant causal links between the genus Ruminococcaceae and fifteen metabolites (Fig. 2): Carnitine levels (OR: 0.89, 95% CI: 0.823–0.979, *P* = .015), isovalerate (i5:0) levels (OR: 1.07, 95% CI: 1.002–1.157, *P* = .041), 2-hydroxyoctanoate levels (OR: 0.84, 95% CI: 0.784–0.917, *P* < .001), 6-hydroxyindole sulfate levels (OR: 0.90, 95% CI: 0.846–0.974, *P* = .007), heptenedioate (C7:1-DC) levels (OR: 1.11, 95% CI: 1.012–1.219, *P* = .026), 4-methylhexanoylglutamine levels (OR: 1.09, 95% CI: 1.001–1.192, *P* = .045), Uridine levels (OR: 1.09, 95% CI: 1.016–1.171, *P* = .016), X-13866 levels (OR: 0.90, 95% CI: 0.843–0.976, *P* = .009), X-17354 levels (OR: 1.11, 95% CI: 1.014–1.214, *P* = .022), X-18345 levels (OR: 1.14, 95% CI: 1.046–1.258, *P* = .003), X-22834 levels (OR: 1.23, 95% CI: 1.136–1.333, *P* < .001), X-25519 levels (OR: 0.86, 95% CI: 0.805–0.929, *P* < .001), serine to pyruvate ratio (OR: 0.86, 95% CI: 0.794–0.940, *P* < .001), uridine to 2’-deoxyuridine ratio (OR: 0.92, 95% CI: 0.861–0.995, *P* = .036), and histidine (His) to glutamine (Gln) ratio (OR: 1.15, 95% CI: 1.070–1.240, *P* < .001) (Supplementary File 2, Supplemental Digital Content, https://links.lww.com/MD/Q797).

### 3.3. The relationship between metabolites and CD

Based on the 15 metabolites mentioned above, we found that all fifteen metabolites have a causal relationship with CD (carnitine levels, OR: 0.79, 95% CI: 0.684–0.914, *P* = .001; isovalerate (i5:0) levels, OR: 1.27, 95% CI: 1.016–1.581, *P* = .035; 2-hydroxyoctanoate levels, OR: 1.15, 95% CI: 1.001–1.328, *P* = .047; 6-hydroxyindole sulfate levels, OR: 0.73, 95% CI: 0.548–0.979, *P* = .036; heptenedioate (C7:1-DC) levels, OR: 1.41, 95% CI: 1.052–1.896, *P* = .022; 4-methylhexanoylglutamine levels, OR: 0.74, 95% CI: 0.598–0.927, *P* = .008; Uridine levels, OR: 0.75, 95% CI: 0.596–0.944, *P* = .014; X-13866 levels, OR: 0.80, 95% CI: 0.644–0.999, *P* = .049; X-17354 levels, OR: 0.76, 95% CI: 0.615–0.942, *P* = .012; X-18345 levels, OR: 1.15, 95% CI: 1.004–1.322, *P* = .044; X-22834 levels, OR: 0.78, 95% CI: 0.639–0.954, *P* = .015; X-25519 levels, OR: 0.75, 95% CI: 0.598–0.929, *P*-value: .009; serine to pyruvate ratio, OR: 1.56, 95% CI: 1.180–2.054, *P* = .002; uridine to 2’-deoxyuridine ratio, OR: 1.14, 95% CI: 1.033–1.259, *P* = .010; His to Gln ratio, OR: 0.80, 95% CI: 0.642–0.990, *P* = .040) (Supplementary File S2, Supplemental Digital Content, https://links.lww.com/MD/Q797).

### 3.4. Reverse Mendelian randomization analysis

To assess potential reverse causation (i.e., the effect of CD on gut microbiota), we performed reverse MR analysis for all gut microbiota identified in our primary analysis. None of the gut microbiota showed statistically significant causal effects (*P* > .05). Key results are summarized in Supplementary File S3 (Supplemental Digital Content, https://links.lww.com/MD/Q797). Briefly, the IVW method yielded non-significant estimates for all taxa (e.g., *Intestinimonas*: β = 0.025, 95% CI: −0.22 to 0.27, *P* = .84; *Streptococcus*: β = 0.017, 95% CI: −0.48 to 0.51, *P* = .73). Sensitivity analyses (weighted median, MR Egger) were consistent with IVW results.

### 3.5. Sensitivity

The intercept of MR-Egger (Supplementary File S4, Supplemental Digital Content, https://links.lww.com/MD/Q797) and MRPRESSO (Supplementary File S5, Supplemental Digital Content, https://links.lww.com/MD/Q797) have been analyzed to ensure the absence of horizontal pleiotropy. The stability of the results is indicated by scatter plots (Fig. S1, Supplemental Digital Content, https://links.lww.com/MD/Q796) and funnel plots (Fig. S2, Supplemental Digital Content, https://links.lww.com/MD/Q796). Therefore, the reliability and validity of the identified causal relationships have been further supported.

### 3.6. The association between gut microbiota and CD mediated by metabolites

We identified 15 metabolites (carnitine levels, isovalerate (i5:0) levels, hydroxyoctanoate levels, 6-hydroxyindole sulfate levels, heptenedioate, 4-methylhexanoylglutamine levels, uridine levels, X-13866 levels, X-17354 levels, X-18345 levels, X-22834 levels, X-25519 levels (Supplementary File S6, Supplemental Digital Content, https://links.lww.com/MD/Q797), Serine to pyruvate ratio, Uridine to 2’-deoxyuridine ratio, and His to Gln ratio) with mediating effects in the impact of genus Ruminococcaceae UCG013 on CD (Table 1).

**Table 1:** The metabolites mediation effect of gut microbiota on CD.*

Mediator	Mediation effect β (95% CI)	Mediated proportion (%)
Carnitine levels	0.0128 (−0.0214, 0.0470)	13.34
Isovalerate (i5:0) levels	0.0003 (−0.0520, 0.0526)	0.33
2-Hydroxyoctanoate levels	−0.0099 (−0.0308, 0.0109)	\
6-Hydroxyindole sulfate levels	0.0039 (−0.0860, 0.0939)	4.15
Heptenedioate (C7:1-DC) levels	0.0288 (−0.0730, 0.1307)	30.10
4-Methylhexanoylglutamine levels	−0.0380 (−0.1035, 0.0274)	\
Uridine levels	0.0076 (−0.0585, 0.0739)	8.00
X-13866 levels	−0.0140 (−0.0625, 0.0345)	\
X-17354 levels	0.0167 (−0.0418, 0.0752)	17.42
X-18345 levels	0.0115 (−0.0097, 0.0328)	12.07
X-22834 levels	−0.0173 (−0.0672, 0.0325)	\
X-25519 levels	0.0351 (−0.0301, 0.1004)	36.69
Serine to pyruvate ratio	0.0111 (−0.1114, 0.1337)	11.59
Uridine to 2’-deoxyuridine ratio	0.0003 (−0.0354, 0.0361)	0.36
Histidine to glutamine ratio	0.0055 (−0.0434, 0.0545)	5.81

CD = Crohn disease, CI = confidence interval.

* Mediated proportions are not reported when the 95% confidence interval for β included zero (indicating non-significant mediation) or when the effect direction was negative. These cases reflect either: a) No statistically significant mediation (CI crosses zero), or β) Model instability from opposing direct/indirect effects (negative values).

## 4. Discussion

Our study aimed to show the cause-and-effect relationship between gut microbiota and CD. We used MR analysis to explore the link between gut microbiota and CD based on existing GWAS data and to determine if metabolites influence the relationship between them. Our results indicated that the genetically predicted Genus Ruminococcaceae UCG013 was linked to a higher risk of CD, and fifteen metabolites influenced this effect.

Ruminococcaceae, one of the 4 family-level taxa distinguishing between lean and obese individuals, is a significant butyrate producer and a crucial bacterium for intestinal health.^[15–17]^ The Ruminococcaceae family includes Genus Ruminococcaceae UCG013, known for its butyrate-producing abilities. In 2 separate longitudinal Chinese cohort studies, Ruminococcaceae UCG013 was more abundant in populations with a diverse diet, particularly among participants who consumed various fruits and dairy products.^[18]^ Zhai et al^[19]^ found that Ruminococcaceae UCG013 could decrease the risk of alcoholic fatty liver disease and nonalcoholic steatohepatitis (NASH) due to the presence of SCFAs, essential metabolites produced by gut microbiota. SCFAs support the health of intestinal cells and play a role in preserving the function of the intestinal barrier.^[20]^ Tu et al^[21]^ also found that Ruminococcaceae UCG013 increases the content of SCFAs and enhances immunity. Hu et al^[22]^ suggested that Ruminococcaceae UCG013 plays a vital role in intestinal protection in mice. Cao et al^[23]^ identified Ruminococcaceae UCG013 as a novel regulator of IBDs via the effects of its metabolites. Li et al’s research^[24]^ suggested that the imbalance of gut microbiota in ulcerative colitis (UC) is related to Ruminococcaceae UCG013. Besides, Ruminococcaceae UCG013 was causally associated with a reduced risk of erectile dysfunction development,^[25]^ urological cancers,^[26]^ metabolic disorders,^[27,28]^ immune thrombocytopenia (ITP) and Henoch-Schönlein purpura.^[29]^ However, there has been no research on the correlation between Ruminococcaceae UCG013 and CD. Our results suggested that Ruminococcaceae UCG013 was a risk factor for CD, which may be influenced by various factors such as host genetics, environmental factors, and microbial interactions. Critically, reverse MR analysis (Supplementary File S3, Supplemental Digital Content, https://links.lww.com/MD/Q797) revealed no significant causal effects of CD on gut microbiota (all *P* > .05), effectively excluding reverse causation as a driver of our observed associations. This discovery provides new clues for further understanding the pathophysiological processes of CD, which may contribute to the development of more accurate disease diagnosis methods and more targeted treatment strategies in the future. Further research is warranted to reveal the exact mechanism of action of Ruminococcaceae UCG013 in intestinal diseases, which may also provide new targets for the future treatment of CD.

The body’s breakdown of hard-to-digest dietary fiber components through the gut microbiota is a primary energy source for the colonic epithelium. It has been shown to play a vital role in maintaining intestinal homeostasis.^[30]^ AAs are essential for intestinal growth, mucosal integrity, and barrier function maintenance. Some of these bacterial metabolites can inhibit the respiration and proliferation of colon epithelial cells, affecting barrier function.^[31]^ Research indicates that AA metabolism significantly influences cellular operations, with immune cells exceptionally responsive to infections and variations in the tissue environment, underscoring a strong dependency on metabolic conditions.^[32]^ The work of Scoville et al supports this perspective,^[33]^ who have shown that specific AA and metabolites related to the tricarboxylic acid cycle exhibit notable alterations in patients with CD. Various indications suggest that changes in AA metabolism and energy homeostasis may play a vital role in the pathogenesis of IBD. An experiment^[34]^ has demonstrated that Gln treatment can reduce endoplasmic reticulum stress and apoptosis in TNBS-induced colitis. Endoplasmic reticulum stress and apoptotic cell death are crucial factors in the development and persistence of IBD. Gln can partially inhibit the expression of the PERK pathway, plague globulin heavy chain binding protein, and caspase-3 induced by Brevidin An and tunicamycin. Regulating the endoplasmic reticulum stress signal and anti-apoptosis may protect Gln against TNBS-induced colitis injury. In vitro, research results indicate that Gln impacts cytokine production by macrophages and lymphocytes.^[35]^ Giriş et al’s experiment^[36]^ demonstrated that treatment with Gln significantly increased heme oxygenase-1 (HO-1) expression and GSH levels while reducing NF-κB expression. The pharmacological induction of HO-1 is associated with the inhibition of inducible nitric oxide synthase activity and the reduction of intestinal injury.^[37]^ These experiments indicate that Gln benefits colitis and exerts regulatory effects through different mechanisms. Benjamin et al^[38]^ conducted a clinical study and found that Gln significantly improves intestinal mucosal permeability and structure. In addition, His also plays a vital role in the intestine. Treatment of Caco-2 cells with His significantly inhibited the transcriptional activity of the I-8 promoter. His transcriptional level inhibited the secretion of I-8 induced by hydrogen peroxide and TNF-α in intestinal epithelial cells.^[39]^ Moreover, His can also serve as a clinical biomarker for the treatment of IBD. Ooi et al^[40]^ conducted a study and suggested that the concentration of His in the plasma of patients in the active phase was significantly reduced compared to patients in remission. His plasma content is closely related to serum C-reactive protein levels and clinical disease activity in patients with UC.

We performed Kyoto Encyclopedia of Genes and Genomes pathway enrichment analysis using MetaboAnalyst 5.0. This analysis revealed significant enrichment (false discovery rate-corrected *P* < .05) for 4 key metabolic pathways: Tryptophan metabolism (involving 6-hydroxyindole sulfate), Pyrimidine metabolism (involving uridine and 2’-deoxyuridine), Fatty acid metabolism (involving carnitine and isovalerate), and AA metabolism (involving serine, pyruvate, His, Gln, and their ratios). These interconnected pathways represent core biological processes relevant to CD pathophysiology, including microbial-host signaling (tryptophan metabolism), nucleotide synthesis and energy transfer (pyrimidine metabolism), mitochondrial energy production (fatty acid metabolism), and mucosal integrity maintenance (AA metabolism). The co-enrichment of these pathways suggests that the mediating effect likely arises from coordinated perturbations in these metabolic networks rather than isolated actions of individual metabolites. This systems-level perspective provides a more integrated understanding of how Ruminococcaceae UCG013 might influence CD risk through metabolite-mediated mechanisms.

This study has some limitations. First, our analysis was based on the European population, which may restrict its generalizability. Second, the GWAS dataset has a limited number of cases, and it is hoped that more comprehensive GWAS data will be available for validation. Third, our study used summary-level statistics rather than individual-level data, preventing further exploring causal links between subgroups such as females and males. Fourthly, the MR analysis relies on European-ancestry GWAS datasets, and thus the causal inference between macrophages and CD may not be generalizable to non-European populations. Future replication studies in Asian cohorts (e.g., Chinese patients) are encouraged to validate these findings. Lastly, we lack experimental validation of the relationship between gut microbiota, metabolites, and CD.

## 5. Conclusion

In conclusion, our study identified a causal relationship between gut microbiota and CD, with a small proportion of the effect mediated by fifteen metabolites. Further research is needed on additional factors as potential mediators.

## Acknowledgments

The authors sincerely thank the authors of the included articles in this review for sharing the relevant data.

## Author contributions

**Conceptualization:** Heng Shi, Qin Peng.

**Data curation:** Heng Shi.

**Formal analysis:** Heng Shi, Qin Peng.

**Funding acquisition:** Heng Shi.

**Investigation:** Heng Shi.

**Methodology:** Heng Shi.

**Project administration:** Heng Shi.

**Resources:** Heng Shi.

**Software:** Heng Shi.

**Supervision:** Heng Shi.

**Validation:** Heng Shi.

**Visualization:** Heng Shi.

**Writing – original draft:** Heng Shi.

**Writing – review & editing:** Heng Shi.

## Supplementary Material




