# Supplementary material for: Role of metabolites in mediating the effect of gut microbiota on Crohn disease: A two-step Mendelian randomization (MR) study
Source: Medicine (Baltimore). 2025 Nov 28;104(48):e46253. doi: 10.1097/MD.0000000000046253 (PMC12662486; doi:10.1097/MD.0000000000046253)

**Supplementary Figure 1**

Scatter plot showed the causal associations between gut microbiota and Crohn's disease.


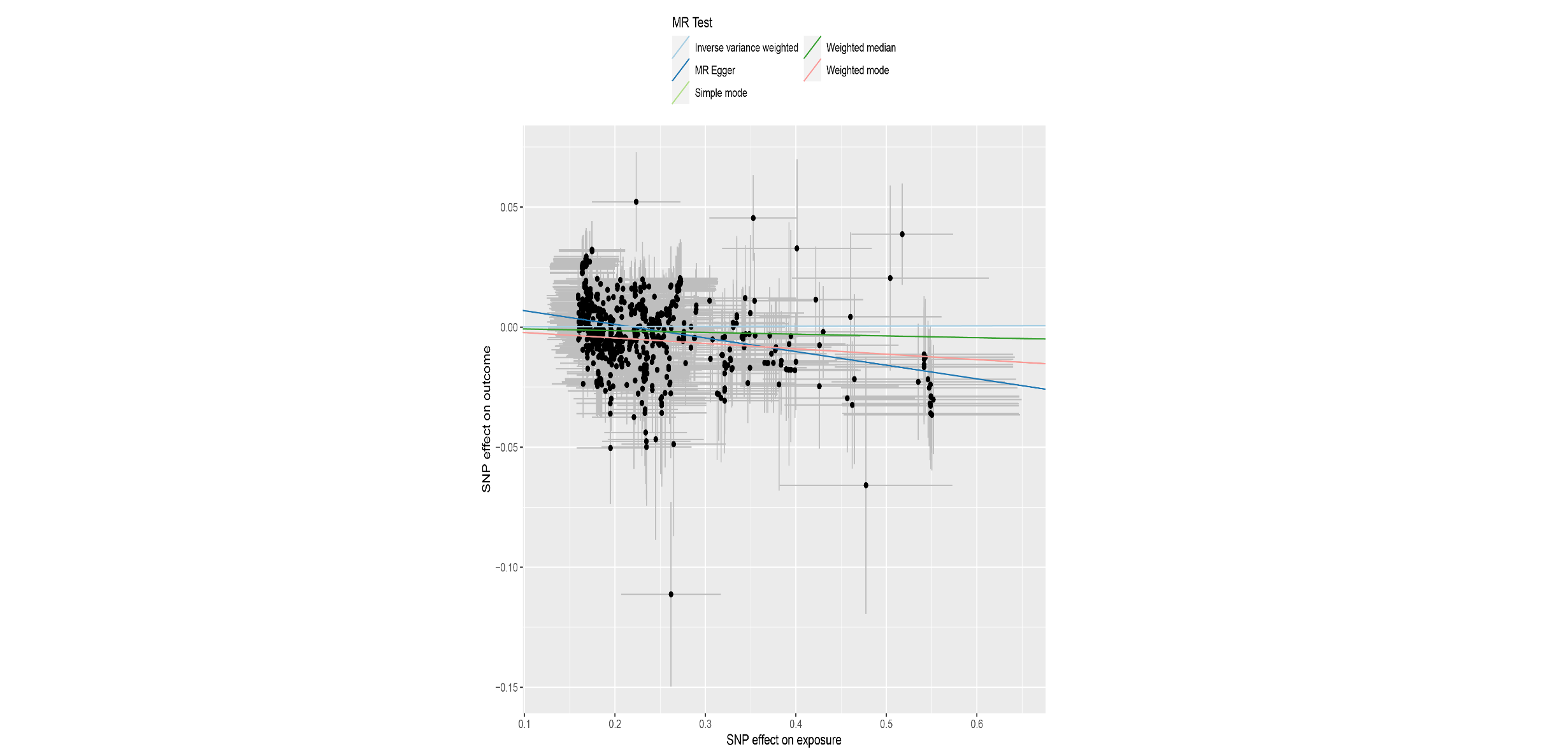


**Supplementary Figure 2**

Funnel plot showed the causal associations between gut microbiota and Crohn's disease.


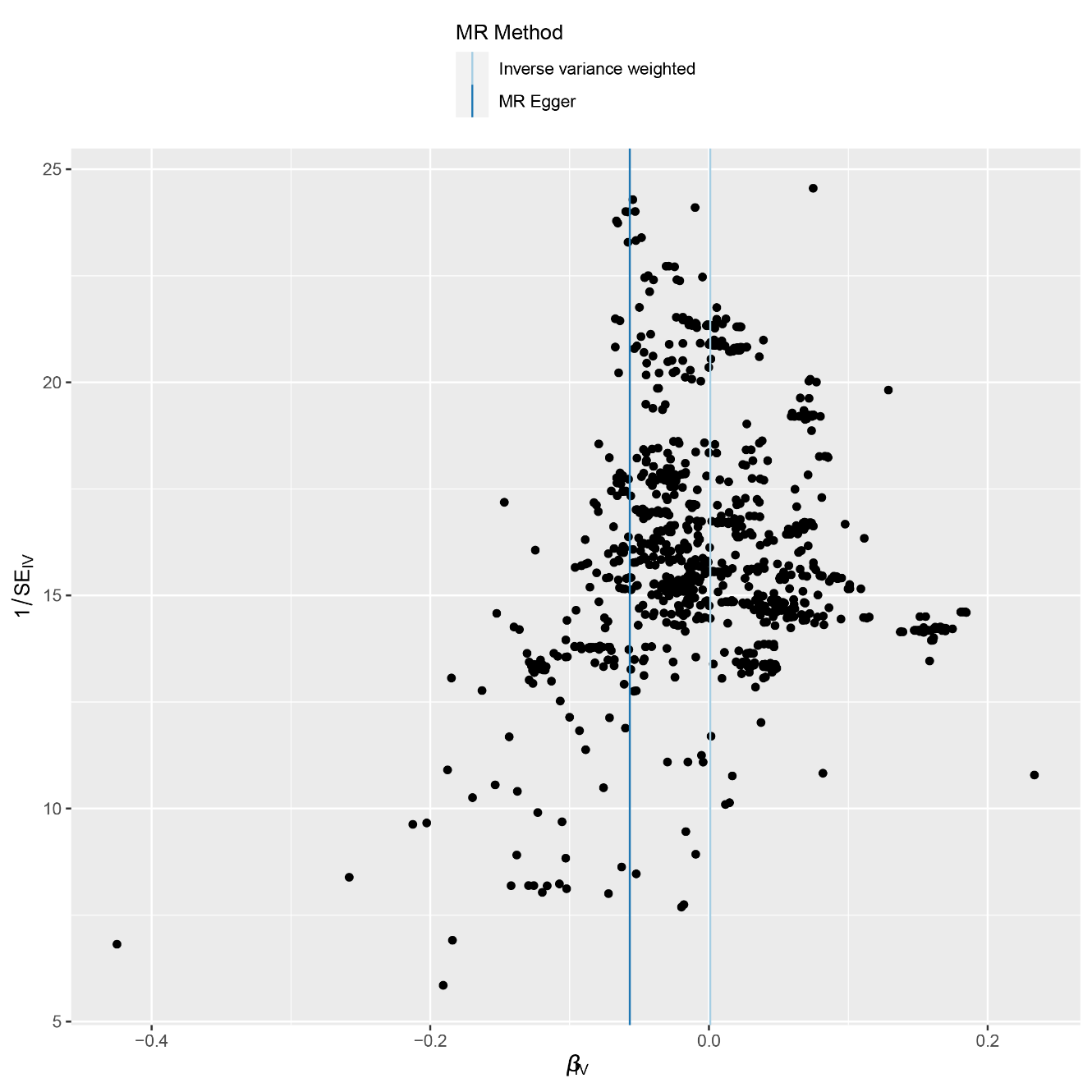

Supplement: Supplementary file 2 [file medi-104-e46253-s002.docx]
